# Professor of Materia Medica in the University of Louisville

**Published:** 1849-07

**Authors:** 


PROFESSOR OF MATERIA MEDICA IN THE UNIVERSITY OF LOUISVILLE.

Lewis Rogers, M.D., has been elected to the chair of Materia Medica and Therapeutics in the University of Louisville made vacant by the resignation of Professor Short. This announcement, we feel quite sure, will be received with pleasure by the friends of the school abroad, as it has been hailed with delight by the community of which Dr. Rogers is a member. Dr. R. has been known to the citizens of

Louisville from his youth up, and has for a number of years been one of the most popular and eminent practitioners in the city. His social qualities render him a worthy successor of a gentleman who is justly esteemed a model for his social virtues; while he brings with him into the school great professional zeal, and experience derived from long and daily intercourse with the sick. No fears are entertained by his friends that he will acquit himself with credit and win additional distinction in the new sphere of duty to which he has been appointed.

